# Characterization of the Complete Chloroplast Genome of *Gaultheria nummularioides* D.Don 1825 (Ericaceae)

**DOI:** 10.12688/f1000research.127937.1

**Published:** 2022-11-21

**Authors:** Xiao-Juan Cheng, Yan-Ling Xu, Catherine M. Bush, Yan-Jun Lin, Xin-Yu Du, Lu Lu

**Affiliations:** 1 School of Pharmaceutical Sciences and Yunnan Key Laboratory of Pharmacology for Natural Products, Kunming Medical University, Kunming, China; 2 Department of Biology, University of North Carolina at Greensboro, Greensboro, NC, USA; 3 Plant Germplasm and Genomics Center, Germplasm Bank of Wild Species, Kunming Institute of Botany, Chinese Academy of Sciences, Kunming, China

**Keywords:** Ericaceae; Gaultheria nummularioides; plastid genome; phylogeny

## Abstract

*Gaultheria nummularioides *D.Don 1825 (Ericaceae) is a traditional Chinese medicinal plant used to treat rheumatoid arthritis. The complete chloroplast genome of *G*. *nummularioides* has been sequenced and assembled. The genome is 176,207 bp in total with one large single copy (LSC: 107,726 bp), one small single copy (SSC: 3,389 bp), and two inverted repeat regions (IRa and IRb; each 32,546 bp). The chloroplast genome encoded a total of 110 unique genes; the GC content of these genes is 36.6%. The results based on phylogenetic analysis confirmed that *G. nummularioides* diverged later than *G.* *praticola*, and the sister-group relationship between *G. nummularioides* and the clade comprising *G.* *fragrantissima *Wall. 1820 and *G.* *hookeri *C.B.Clarke 1882 was strongly supported, revealing the phylogenetic position of *G*. *nummularioides*. This work provides additional information for studying the genetic diversity of *G*. *nummularioides* and its closely related taxa, as well as further exploration of chloroplast genomes in the Ericaceae family.

## Introduction

As a traditional medicinal plant, *Gaultheria nummularioides* D. Don 1825 is often used to treat rheumatoid arthritis ( Luo et al. 2018). This species is a small shrub predominantly distributed in alpine meadows from 1300 to 4600 m in China (mainly in the Himalaya-Hengduan Mountains region (HHM) in Yunnan, Sichuan, and Tibet), as well as in other regions in Southeast Asia ( Fritsch et al. 2008). It is morphologically distinct from most other species from the HHM region based on characteristics such as growth habit and leaf and corolla indumentum ( Middleton 1991). Middleton (1991) therefore placed *G. nummularioides* into a separate section, i.e., sect. *Monoanthemona* Middleton from ser. *Leucothoides* (Airy-Shaw) Middleton of sect. *Brossaea* (L.) Middleton. Although *G.* *nummularioides* did fall within the Leucothoides clade within Gaultherieae, it is not a monophyletic species, as its evolution may have involved gene introgression or gene capture based on four cpDNA genic regions ( *mat*K, *rpl*16, *trn*L- *trn*F, and *trn*S- *trn*G) indicated one sample of *G. nummularioides* from Bhutan diverged earlier but other five samples from China later than those of *G. praticola* C.Y. Wu 1981 ( Lu et al. 2010). Chloroplast DNA is mostly maternally inherited and is characterized by a relatively small genome and slow mutation rate, which make it extremely useful in inferring phylogeny ( Palmer et al. 1988). Therefore, the complete chloroplast genome is of great value for understanding the phylogenetic relationships and maternal inheritance within the species and closely related taxa ( Jung et al. 2014; Xu et al. 2021). However, the sequence and characteristics of *G.* *nummularioides* had not been identified. This current study presents the complete chloroplast genome of *G. nummularioides* and its resulting phylogenetic relationship.

## Methods

The sample was collected from Duoxiongla in Motuo County, Tibet, China (29°30′37″N, 94°51′25″E). The voucher specimen was deposited at the herbarium of the Kunming Institute of Botany (collection number: LL-07304; contact person: Lu Lu, lulukmu@163.com; https://www.cvh.ac.cn/spms/detail.php?id=ea952b43) under the voucher number 1248321. The plants were collected and studied in accordance with the regulations of the author's institution and national or international regulations; no specific permits were required.

Genomic DNA was extracted from leaves dried with silica gel using the CTAB method ( Doyle and Doyle 1987). The raw reads were obtained by next-generation sequencing, conducted on the Illumina Solexa platform (Illumina, New England Biolabs). The clean reads were *de novo* assembled (a assemble method for constructing genomes without a reference sequence) using GetOrganelle v1.7.5.0 ( Jin et al. 2020). Bandage v0.8.1 ( Wick et al. 2015) was used for visualization of the assembly results. Annotation of the genome was conducted and then manually modified by Geneious v8.0.2 (Biomatters Ltd., Auckland, New Zealand). OrganellarGenomeDRAW v1.3.1 ( Greiner et al. 2019) was used to draw the chloroplast genome map. The complete chloroplast genome of *G. nummularioides* and 11 complete chloroplast genomes of related species from the genus *Gaultheria*, in addition to those of four samples as outgroup from the Vaccinieae tribe of Ericaceae were aligned using HomBlocks v1.0 ( Bi et al. 2018). A maximum likelihood phylogenetic tree was reconstructed by RAxML v8.2.X ( Stamatakis 2014) with GTRGAMMA substitution model and 1000 rapid bootstrap replicates.

## Results

**Figure 1.**
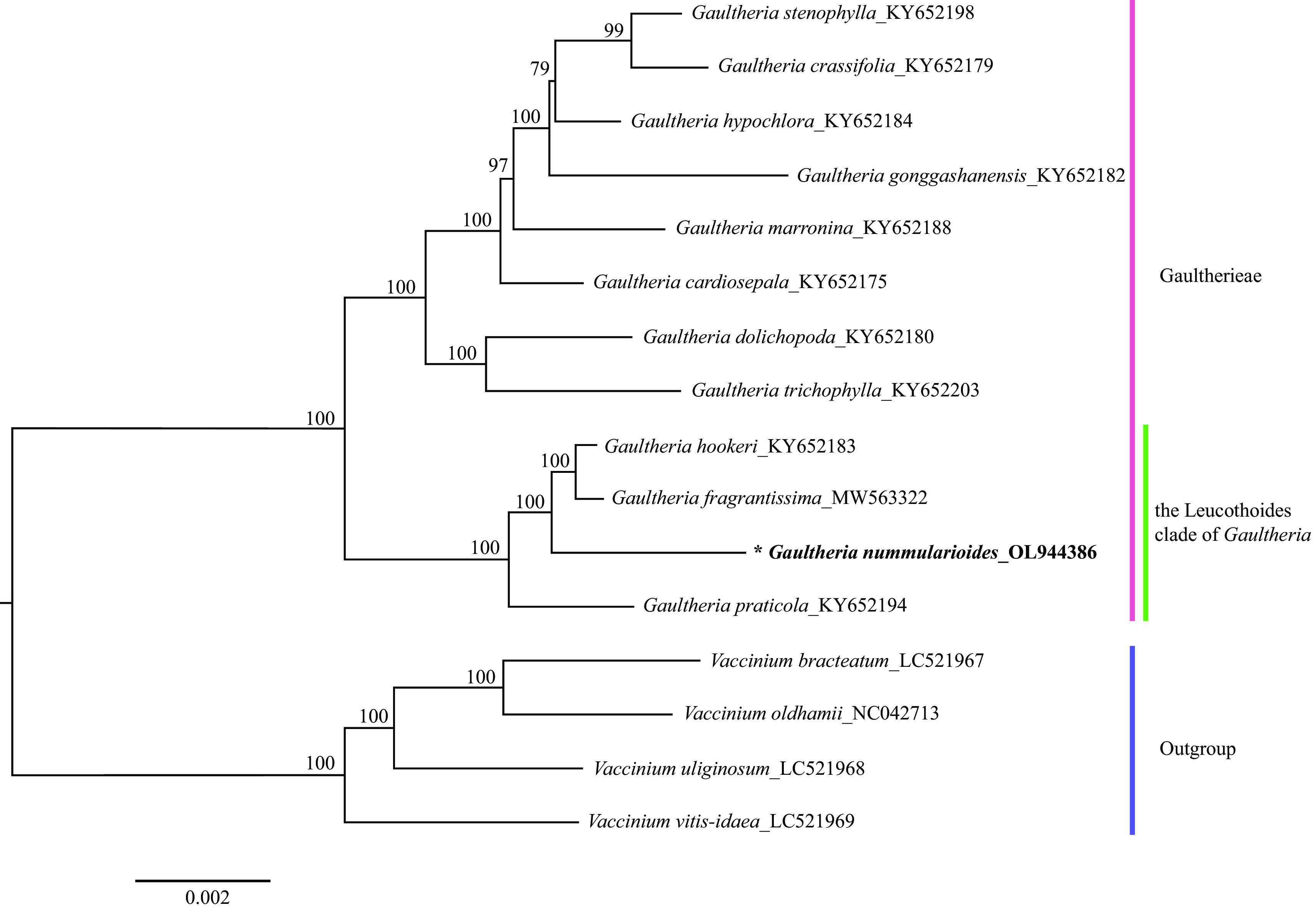
Maximum likelihood tree based on 16 complete chloroplasts genomes of Ericaceae, including four outgroup species. GenBank accession numbers are listed beside the Latin name. The bootstrap support values based on 1000 replicates are shown next to the nodes. *Gaultheria nummularioides* is marked in bold and with an asterisk.

The complete chloroplast genome of *G. nummularioides* (GenBank accession no. OL944386) is a typical quadripartite structure with 176,207 bp in total length, and is composed of a large single-copy (LSC: 107,726 bp) region, a small single-copy (SSC: 3,389 bp) region, and a pair of inverted repeats (IRs: 32,546 bp). The GC content in the chloroplast genome is 36.6%. This chloroplast genome encoded a total of 110 unique genes, of which 25 were duplicated once in the IR regions. Three genes were characterized by their multiple duplicates: *rpl*23 and *rps*14 have three duplicates and *trnf*M has four duplicates in both LSC and IR regions. Out of the 110 genes, there were 76 protein-coding, 30 tRNA, and 4 rRNA genes. The results based on maximum likelihood analysis confirmed that *G. nummularioides* diverged later than *G. praticola*, the sister-group relationship between *G. nummularioides* and the clade comprising *G. fragrantissima* Wall. 1820 and *G. hookeri* C.B.Clarke 1882 was strongly supported ( Figure 1). The four taxa comprise the Leucothoides clade.

## Data Availability

GenBank: Gaultheria nummularioides chloroplast, complete genome, Accession number OL944386: https://identifiers.org/ncbi/insdc:OL944386. BioProject: Gaultheria nummularioides Raw sequence reads, Accession number PRJNA744357: https://identifiers.org/NCBI/bioproject:PRJNA744357. SRA: The complete chloroplast genome: Gaultheria nummularioides, Accession number SRX11377412: https://www.ncbi.nlm.nih.gov/sra/?term=SRX11377412. Bio-Sample: Plant sample from Gaultheria nummularioides, Accession number SAMN20088414: https://identifiers.org/biosample:SAMN20088414.
