# Characterization of the Complete Chloroplast Genome of *Gaultheria nummularioides* D.Don 1825 (Ericaceae)

**DOI:** 10.12688/f1000research.127937.2

**Published:** 2023-09-04

**Authors:** Xiao-Juan Cheng, Yan-Ling Xu, Catherine M. Bush, Yan-Jun Lin, Xin-Yu Du, Lu Lu

**Affiliations:** 1 School of Pharmaceutical Sciences and Yunnan Key Laboratory of Pharmacology for Natural Products, Kunming Medical University, Kunming, China; 2 Department of Biology, University of North Carolina at Greensboro, Greensboro, NC, USA; 3 Plant Germplasm and Genomics Center, Germplasm Bank of Wild Species, Kunming Institute of Botany, Chinese Academy of Sciences, Kunming, China

**Keywords:** Ericaceae; Gaultheria nummularioides; plastid genome; phylogeny

## Abstract

*Gaultheria nummularioides *D.Don 1825 (Ericaceae) is a traditional Chinese medicinal plant used to treat rheumatoid arthritis. The complete chloroplast genome of *G*. *nummularioides* has been sequenced and assembled. The genome is 176,207 bp in total with one large single copy (LSC: 107,726 bp), one small single copy (SSC: 3,389 bp), and two inverted repeat regions (IRa and IRb; each 32,546 bp). The chloroplast genome encoded a total of 110 unique genes; the GC content of these genes is 36.6%. The results based on phylogenetic analysis of the complete chloroplast genome suggests that *G. nummularioides* diverged later than *G.* *praticola*, the sister relationship between *G. nummularioides* and the clade comprising *G. fragrantissima *Wall. 1820 and *G. hookeri *C.B. Clarke 1882 was strongly supported. This study provides additional information on the genetic diversity of *G*. *nummularioides*, its closely related taxa, and further exploration of chloroplast genomes in the Ericaceae family.

## Introduction

As a traditional medicinal plant, *Gaultheria nummularioides* D. Don 1825 is often used to treat rheumatoid arthritis ( Luo et al. 2018). This species is a small shrub predominantly distributed in alpine meadows from 1300 to 4600 m in China (mainly in the Himalaya-Hengduan Mountains region [HHM] in Yunnan, Sichuan, and Tibet), as well as in other regions in Southeast Asia ( Fritsch et al. 2008). It is morphologically distinct from most other *Gaultheria* species from the HHM region based on characteristics such as growth habit and leaf and corolla indumentum ( Middleton 1991). Middleton (1991) therefore placed *G. nummularioides* into a separate section, i.e., sect. *Monoanthemona* Middleton from ser. *Leucothoides* (Airy-Shaw) Middleton of sect. *Brossaea* (L.) Middleton. Although *G.* *nummularioides* did fall within the Leucothoides clade within Gaultherieae, it is not a monophyletic species, as its evolution may have involved gene introgression or gene capture based on four cpDNA genic regions ( *mat*K, *rpl*16, *trn*L- *trn*F, and *trn*S- *trn*G) indicated one sample of *G. nummularioides* from Bhutan diverged earlier but other five samples from China later than those of *G. praticola* C.Y. Wu 1981 ( Lu et al. 2010). Chloroplast DNA is typically maternally inherited and is characterized by a relatively small genome and slow mutation rate, so the complete chloroplast genome is of great value for understanding the phylogenetic relationships and maternal inheritance within the species and closely related taxa ( Palmer et al. 1988; Jung et al. 2014; Xu et al. 2021). However, the sequence and characteristics of *G.* *nummularioides* had not been sequenced. This study presents the complete chloroplast genome of *G. nummularioides* and its resulting phylogenetic relationship.

## Methods

The sample (Collected on August 3, 2007) was collected from Duoxiongla in Motuo County, Tibet, China (29°30′37″N, 94°51′25″E). The voucher specimen was deposited at the herbarium of the Kunming Institute of Botany (collection number: LL-07304; contact person: Lu Lu, lulukmu@163.com; https://www.cvh.ac.cn/spms/detail.php?id=ea952b43) under the voucher number 1248321. The plants were collected and studied in accordance with the regulations of the author's institution and national or international regulations; no specific permits were required.

After collecting fresh leaves in the field, they were dried using silica gel with discoloration. Subsequently, genomic DNA was extracted using the CTAB method ( Doyle and Doyle 1987). Specifically, 1000 μL of 4×CTAB solution, preheated at 65°C and containing a small amount of mercaptoethanol, was added to ground leaves. Sequencing was carried out on the Illumina HiSeq 2000 platform, generating 2-3 Gb of paired-end reads with a length of 150 bp for each sample. The raw data obtained from sequencing can contain low-quality data or data with connectors. To ensure the quality of subsequent information analysis, the raw data was filtered to eliminate these low-quality data or paired reads with connectors. The specific characteristics of the data to be filtered and removed are as follows: (1) Single-end sequencing reads with an N content exceeding 10% of the read length and (2) Single-end sequencing reads with a number of low-quality bases (Q ≤ 5) that exceeded 50% of the read length. The resulting filtered data is referred to as clean data and is used for subsequent analyses.

**Figure 1.**
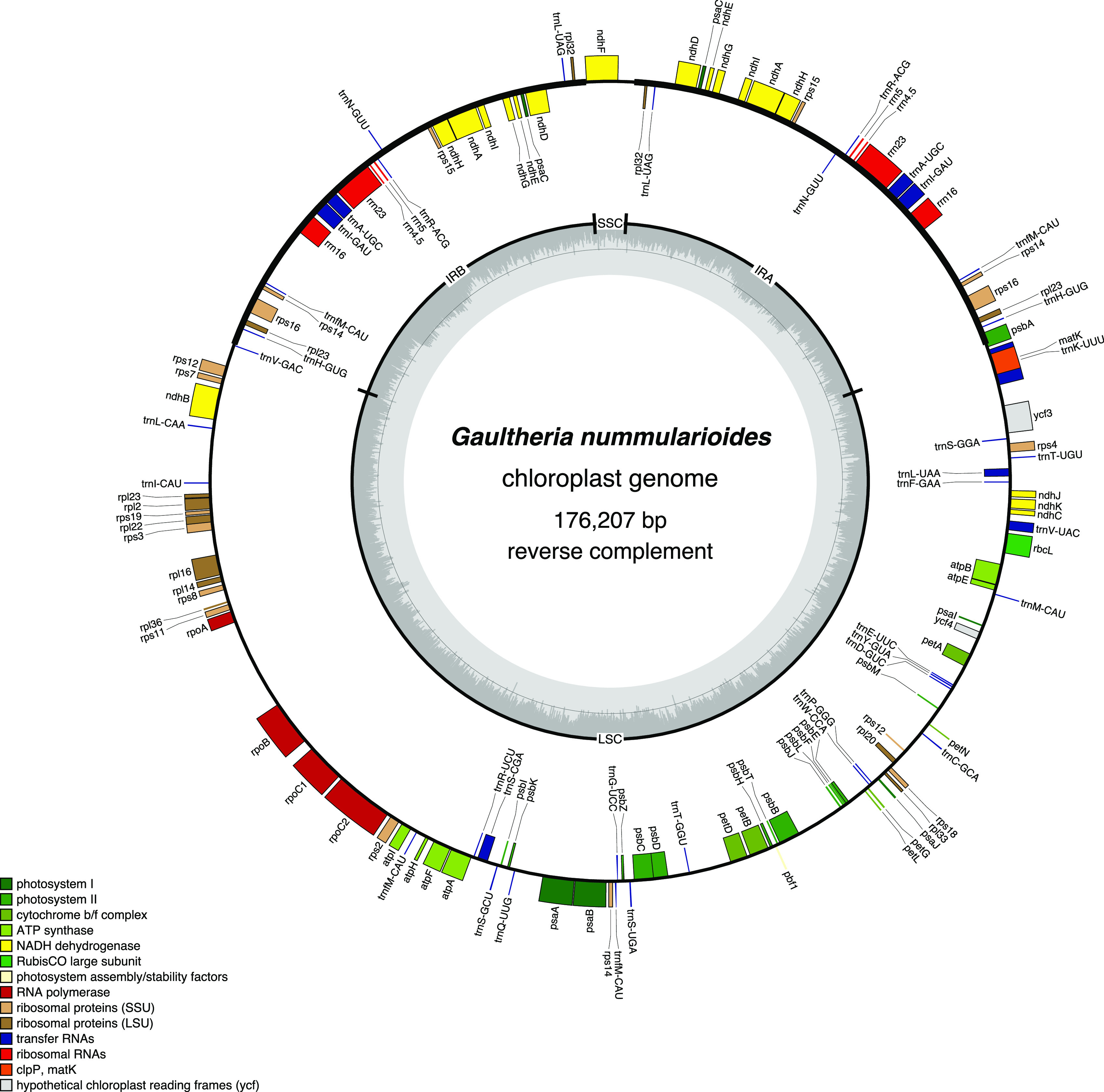
Chloroplast genome map of *Gaultheria nummularioides*.

The clean reads were *de novo* assembled (a assemble method for constructing genomes without a reference sequence) using GetOrganelle v1.7.5.0 ( Jin et al. 2020). Bandage v0.8.1 was used for visualization of the assembly results ( Wick et al. 2015). Annotation of the genome was conducted and then manually modified by Geneious v8.0.2 (Biomatters Ltd., Auckland, New Zealand). OrganellarGenomeDRAW v1.3.1 was used to draw the chloroplast genome map ( Figure 1, Greiner et al. 2019). The complete chloroplast genome of *G. nummularioides*, eleven complete chloroplast genomes of related species from the genus *Gaultheria*, and four samples from the Vaccinieae tribe of Ericaceae as outgroup were aligned using HomBlocks v1.0 ( Bi et al. 2018). A maximum likelihood phylogenetic tree was reconstructed by RAxML v8.2.X with GTRGAMMA substitution model and 1000 rapid bootstrap replicates ( Stamatakis 2014).

## Results

**Figure 2.**
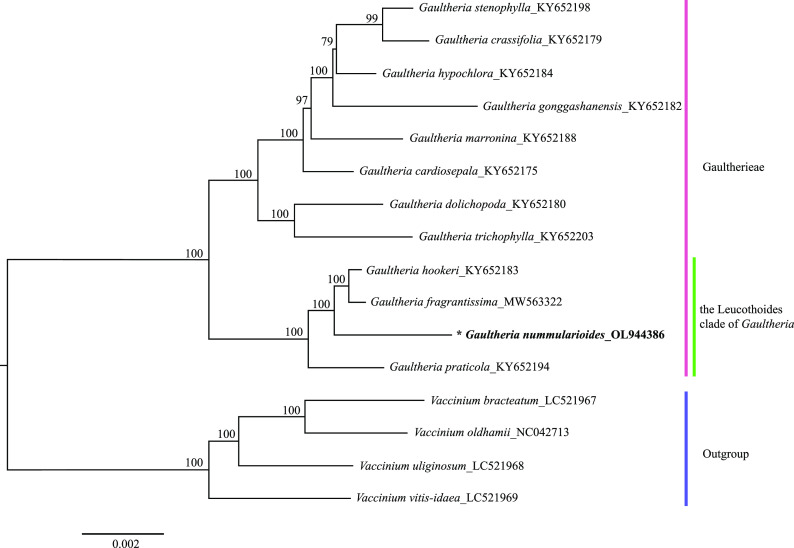
Maximum likelihood tree based on 16 complete chloroplasts genomes of Ericaceae, including four outgroup species. GenBank accession numbers are listed beside the Latin name. The bootstrap support values based on 1000 replicates are shown next to the nodes. *Gaultheria nummularioides* is marked in bold and with an asterisk.

The complete chloroplast genome of *G. nummularioides* (GenBank accession no. OL944386) is a typical quadripartite structure with a total length of 176,207 bp. It is composed of a large single-copy (LSC: 107,726 bp) region, a small single-copy (SSC: 3,389 bp) region, and a pair of inverted repeats (IRs: 32,546 bp). The GC content in the chloroplast genome is 36.6%. This chloroplast genome encoded a total of 110 unique genes, of which 25 were duplicated once in the IR regions. Three genes were characterized by their multiple duplicates: *rpl*23 and *rps*14 have three duplicates each and *trnf*M has four duplicates in both LSC and IR regions. Out of the 110 genes, there were 76 protein-coding, 30 tRNA, and 4 rRNA genes. Maximum likelihood (ML) analysis results show that *G. nummularioides* diverged later than *G. praticola*. The results strongly support the sister-group relationship between *G. nummularioides* and the clade comprising *G. fragrantissima* Wall. 1820 and *G. hookeri* C.B.Clarke 1882 ( Figure 2). The four taxa comprise the Leucothoides clade.

## Data Availability

GenBank: Gaultheria nummularioides chloroplast, complete genome, Accession number OL944386: https://identifiers.org/ncbi/insdc:OL944386. BioProject: Gaultheria nummularioides Raw sequence reads, Accession number PRJNA744357: https://identifiers.org/NCBI/bioproject:PRJNA744357. SRA: The complete chloroplast genome: Gaultheria nummularioides, Accession number SRX11377412: https://www.ncbi.nlm.nih.gov/sra/?term=SRX11377412. Bio-Sample: Plant sample from Gaultheria nummularioides, Accession number SAMN20088414: https://identifiers.org/biosample:SAMN20088414.
