# Supplementary material for: Characterization of the Complete Chloroplast Genome of Gaultheria nummularioides D.Don 1825 (Ericaceae)
Source: F1000Res. 2023 Sep 4;11:1358. Originally published 2022 Nov 21. [Version 2] doi: 10.12688/f1000research.127937.2 (PMC10521052; doi:10.12688/f1000research.127937.2)
Supplement: Peer review history

# Reviewer report: Reviewer response for version 2

**DOI:** 10.5256/f1000research.155153.r203746

**Published:** 2023-09-14

**Authors:** Xing Huang

**Affiliations:** 1 Environment and Plant Protection Institute, Chinese Academy of Tropical Agricultural Sciences, Haikou, China

The authors have addressed most of the comments and revised the manuscript accordingly. I don't have new questions.

Are the datasets clearly presented in a usable and accessible format, and the assembly and annotation available in an appropriate subject-specific repository?

Yes

Are sufficient details of the sequencing and extraction, software used, and materials provided to allow replication by others?

Partly

Are the rationale for sequencing the genome and the species significance clearly described?

Yes

Are the protocols appropriate and is the work technically sound?

Yes

Reviewer Expertise:

Genetics and molecular biology of plants

I confirm that I have read this submission and believe that I have an appropriate level of expertise to confirm that it is of an acceptable scientific standard.

# Reviewer report: Reviewer response for version 2

**DOI:** 10.5256/f1000research.155153.r203745

**Published:** 2023-09-05

**Authors:** Hoang Dang Khoa Do

**Affiliations:** 1 Nguyen Tat Thanh University, Ho Chi Minh City, Ho Chi Minh, Vietnam

Dear Authors,

 Thank you very much for your revised article which was modified based on the reviewer's comments. I do not have any questions for the version 2.

 Best regards.

Are the datasets clearly presented in a usable and accessible format, and the assembly and annotation available in an appropriate subject-specific repository?

Yes

Are sufficient details of the sequencing and extraction, software used, and materials provided to allow replication by others?

Yes

Are the rationale for sequencing the genome and the species significance clearly described?

Partly

Are the protocols appropriate and is the work technically sound?

Yes

Reviewer Expertise:

Genomics, Phylogeny

I confirm that I have read this submission and believe that I have an appropriate level of expertise to confirm that it is of an acceptable scientific standard.

# Reviewer report: Reviewer response for version 1

**DOI:** 10.5256/f1000research.140483.r167786

**Published:** 2023-03-29

**Authors:** Hoang Dang Khoa Do

**Affiliations:** 1 Nguyen Tat Thanh University, Ho Chi Minh City, Ho Chi Minh, Vietnam

Thank you very much for studying on chloroplast genome of *Gaultheria nummularioides *which provides new information about genomic data of *Gaultheria *as well as the Ericaceae family. The content of the manuscript is suitable for a Genome Note of F1000Research.

 I have some comments as the following:

1. Please ask an English editing service for checking the writing of the manuscript. Some sentences are very long.
2. Please provide more details of the sequencing process (i.e., single-end or paired-reads, size of reads 150 bp, 250 bp or 300 bp).
3. Please provide details on the step of filtering raw reads to get clean reads for *de novo* assembly.
4. Please use italic font for species names in the Data availability part.

Thank you.

Are the datasets clearly presented in a usable and accessible format, and the assembly and annotation available in an appropriate subject-specific repository?

Yes

Are sufficient details of the sequencing and extraction, software used, and materials provided to allow replication by others?

Yes

Are the rationale for sequencing the genome and the species significance clearly described?

Partly

Are the protocols appropriate and is the work technically sound?

Yes

Reviewer Expertise:

Genomics, Phylogeny

I confirm that I have read this submission and believe that I have an appropriate level of expertise to confirm that it is of an acceptable scientific standard, however I have significant reservations, as outlined above.

# Response

**Published:** 2023-08-25

**Authors:** Xiaojuan Cheng

**Affiliations:** Kunming Medical University, China

**Question 1**: Please ask an English editing service for checking the writing of the manuscript. Some sentences are very long.

**Response**: One of our co-author, Catherine M. Bush is a native speaker of English from USA and published many papers in English. English has been polished by her.

**Question 2**: Please provide more details of the sequencing process (i.e., single-end or paired-reads, size of reads 150 bp, 250 bp or 300 bp).

**Response**: Sequencing was carried out on the Illumina HiSeq 2000 platform, generating 2-3 Gb of paired-end reads with a length of 150 bp for each sample.

**Question** **3**: Please provide details on the step of filtering raw reads to get clean reads for de novo assembly.

**Response**: The raw data obtained from sequencing may contain low-quality data or data with connectors. To ensure the quality of subsequent information analysis, it is necessary to eliminate these low-quality data or paired reads with connectors, referred to as filtering the raw data. The specific characteristics of the data to be filtered and removed are as follows: (1) Single-end sequencing reads with an N content exceeding 10% of the read length. (2) Single-end sequencing reads with a number of low-quality bases (Q≤5) exceeding 50% of the read length. The resulting filtered data is referred to as clean data and is used for subsequent analyses.

**Question** **4**: Please use italic font for species names in the Data availability part.

**Response**: We correct that in the latest version.

# Reviewer report: Reviewer response for version 1

**DOI:** 10.5256/f1000research.140483.r160447

**Published:** 2023-01-23

**Authors:** Xing Huang

**Affiliations:** 1 Environment and Plant Protection Institute, Chinese Academy of Tropical Agricultural Sciences, Haikou, China

The manuscript has assembled and characterized the chloroplast genome of *Gaultheria nummularioides*, which is meaningful to Ericaceae family. However, several major concerns should be addressed:

1. In methods part, how was the dried leaves processed for DNA isolation by CTAB method? Which should be described.
2. The Illumina Solexa platform was used according to the methods, which is different from the SRA information. The authors should thoroughly check it.
3. How were the clean reads generated? Raw reads were directly generated by the sequencing machine.
4. In methods part, ‘OrganellarGenomeDRAW v1.3.1 (Greiner *et al.* 2019) was used to draw the chloroplast genome map’. And where is the map presented in the manuscript?
5. The sequence of *Gaultheria griffithiana* (NC_057623.1) should be added in phylogenetic analysis.
6. The details of sequencing data should be described in results part.
7. The tRNA and rRNA couldn’t be considered as unique gene.

Are the datasets clearly presented in a usable and accessible format, and the assembly and annotation available in an appropriate subject-specific repository?

Yes

Are sufficient details of the sequencing and extraction, software used, and materials provided to allow replication by others?

Partly

Are the rationale for sequencing the genome and the species significance clearly described?

Yes

Are the protocols appropriate and is the work technically sound?

Yes

Reviewer Expertise:

Genetics and molecular biology of plants

I confirm that I have read this submission and believe that I have an appropriate level of expertise to confirm that it is of an acceptable scientific standard, however I have significant reservations, as outlined above.

# Response

**Published:** 2023-08-25

**Authors:** Xiaojuan Cheng

**Affiliations:** Kunming Medical University, China

**Question** **1**: In methods part, how was the dried leaves processed for DNA isolation by CTAB method? Which should be described.

**Response**: After collecting fresh leaves in the field, they were dried using silica gel with discoloration. Subsequently, genomic DNA was extracted using the CTAB method (Doyle and Doyle, 1987). Specifically, 1000 μL of 4× CTAB solution, preheated at 65°C and containing a small amount of mercaptoethanol, was added to leaf grinding.

**Question** **2**: The Illumina Solexa platform was used according to the methods, which is different from the SRA information. The authors should thoroughly check it.

**Response**: Upon further review, the sequencing methodology utilizes the Illumina HiSeq 2000 platform and has been rectified.

**Question** **3: **How were the clean reads generated? Raw reads were directly generated by the sequencing machine.

**Response**: Raw data obtained from sequencing contains some low-quality data or data with connectors. In order to ensure the quality of subsequent information analysis, it is necessary to remove these low-quality data or paired reads with connectors, i.e., to filter the raw data. The specific characteristics of the data to be filtered and removed are as follows: (1) the N content in single- end sequencing reads exceeds 10% of the length of the reads; (2) the number of low-quality (Q≤5) bases in single-end sequencing reads exceeds 50% of the length of the reads. The filtered data are called clean data and are used for subsequent analyses.

**Question** **4: **In methods part, ‘OrganellarGenomeDRAW v1.3.1 (Greiner et al. 2019) was used to draw the chloroplast genome map’. And where is the map presented in the manuscript?

**Response**: Thank you for the reminder! We add the map in the latest version.

**Question** **5: **The sequence of *Gaultheria griffithiana* (NC_057623.1) should be added in phylogenetic analysis.

**Response**: This manuscript focuses on reporting the chloroplast genome structure of a single species within the genus *Gaultheria*. We have another manuscript for displaying the plastid phylogenomic relationships of the whole clade (ca. 60 species) within *Gaultheria* at species level (unpublished data). Add one more species cannot help on resolving the phylogenetic position of this species in such a large clade.

**Question** **6: **The details of sequencing data should be described in results part.

**Response**: we reference to the similar description of the other articles and revise it accordingly.

**Question** **7: **The tRNA and rRNA couldn’t be considered as unique gene.

**Response**: We know each of tRNA gene or rRNA gene has two copies in the IR region. The “unique gene” we used in our study means that tRNA and rRNA have not appeared in any other place but in the IR region. We described this in the way following the literature such as Henriquez et al. (2020) and (Gao et al., 2023). We revised this accordingly.

 (a) The genomes contain 114 unique genes, including four rRNA genes, 80 protein-coding genes, and 30 tRNA genes (Henriquez et al., 2020).

 (b) The chloroplast genomes of *R. rubiginosa*, *R. hybrida*, and *R. acicularis* were conserved and contained 115 unique genes, of which 80 were protein-coding genes, 31 were transfer RNA (tRNA) genes, and 4 were ribosomal RNA (rRNA) genes (Gao et al., 2023).

**Reference:**

 Henriquez, C. L., Abdullah, Ahmed, I., Carlsen, M. M., et al. 2020. Molecular evolution of chloroplast genomes in Monsteroideae (Araceae). Planta, 251, 1–16.

 Gao, C., Li, T., Zhao, X., et al. 2023. Comparative analysis of the chloroplast genomes of Rosa species and RNA editing analysis. BMC Plant Biology, 23(1), 318.
